# Exploring the impact of climate change on respiratory health in Nigeria: a scoping review of current research, government policies and programs

**DOI:** 10.1007/s10584-025-03880-0

**Published:** 2025-02-17

**Authors:** Faatihah Niyi-Odumosu, B. Ozoh, Victor Oloruntoba Ope, Boni M. Ale, Olayemi Akinnola, Alexander Iseolorunkanmi, Davies Adeloye

**Affiliations:** 1 School of Applied Sciences, University of the West of England, Bristol, UK; 2 Department of Medicine, Faculty of Clinical Sciences, College of Medicine, University of Lagos, Lagos, Nigeria; 3 Department of Sociology, Covenant University, Ota, Nigeria; 4 Cardiovascular Research Unit, University of Abuja and University of Abuja Teaching Hospital, Gwagwalada Abuja, Nigeria; 5 Institute of Tropical and Infectious Diseases, University of Nairobi, Nairobi, Kenya; 6 Holo Global Health Research Institute, Nairobi, Kenya; 7 Health Data Acumen, Nairobi, Kenya; 8 Department of Biological Sciences, Covenant University, Ota, Nigeria; 9 Covenant University Medical Centre, Covenant University Covenant University, Ota, Nigeria; 10 School of Health & Life Sciences, Teesside University, MiddlesbroughTS1 3BX, UK

**Keywords:** Keywords Climate change, Respiratory health, Nigeria, Policies

## Abstract

Climate change significantly impacts health globally, especially in densely populated, rapidly industrialising and ecologically diverse countries like Nigeria. We analysed climate change policies, studies, programs, and events at the national and subnational levels in Nigeria and explored their effects on public and respiratory health. Using the Preferred Reporting Items for Systematic reviews and Meta-Analyses extension for Scoping Reviews (PRISMAScR) checklist, we searched PubMed, African Journals Online (AJoL), Google Scholar and government data repositories on January 10, 2024. We synthesised results using an adapted sector-level framework based on the World Health Organization (WHO) guidelines. Our searches returned 262 items, of which 32, including research studies, reports and grey documents, were retained for synthesis. Although some policies and programmes, like the Climate Change Act and Nigerian Climate and Health Observatory, exist, implementation is limited across many settings. Key reported respiratory pollutants in Nigeria include particulate matter (PM2.5, PM10), gaseous emissions (CO, SO_2_, NOx), agricultural by-products (NH_3_, H_2_S), greenhouse gases (CH_4_, CO_2_), and microbial contaminants, which collectively increase the risk of respiratory inflammation, infections, and exacerbations of chronic respiratory symptoms and diseases. Our findings underscore a clear link between climate change and worsening respiratory health in many Nigerian settings. The current policies and programmes’ have limited impact, calling for comprehensive reforms, including improved enforcement and targeted action against major pollution sources, recognition of environmental rights, and stronger public health initiatives and community action.

## 1 Introduction

Climate change, which refers to the long-term shifts in weather patterns and temperatures that are caused by human activity or natural variability, is now a global health risk (United Nations 2024). Driven mostly by greenhouse gas emissions from burning fossil fuels like coal, oil, and gas, it has remained a subject of debate among world leaders and organisations. The 2015 United Nations (UN) Climate Change Conference (COP21) Paris agreement focused on limiting global warming to 1.5 °C by the end of this century (Zhang et al. 2018; WHO and UN FCCC 2015). Although many low- and middle-income countries (LMICs), including African countries like Nigeria, appear to contribute less to global emissions, they experience the most severe impacts and consequences (Javadinejad et al. 2019; D’Amato et al. 2014). These include altered disease patterns, threats to food and water security, and frequent extreme weather events and environmental changes (Javadinejad et al. 2019; D’Amato et al. 2014). Specifically, increase in the levels of air pollution and thermal conditions, both contributing to the global disease burden from respiratory illnesses, have continued to be reported (Opoku et al. 2021; Adelekan and Fregene 2015). These impacts necessitate urgent policy interventions for adaptation and support for vulnerable populations (Javadinejad et al. 2019).

The connection between climate change and increased risk of respiratory diseases is well-established (Adeloye et al. 2022). Air pollution and extreme heat aggravate existing respiratory diseases such as asthma and chronic obstructive pulmonary disease (COPD) and are linked to the development of other diseases, including lung cancer (Pona et al. 2021; Yusuf et al. 2023). Nigeria represents an important focus for strategic intervention globally, considering her increasing population, currently estimated at about 230 million (about a quarter of the African population) (NBS 2023). Ensuing anthropogenic human activities continue to drive increased climate risks (Afinotan 2022; Ajibade et al. 2013; Akpodiogaga-a and Odjugo 2010; Ilevbare 2019), which exacerbate health vulnerabilities, particularly respiratory disorders (Eludoyin et al. 2014; WHO and UN FCCC 2015). For example, Nigeria’s gas flaring alone contributes more greenhouse gases than the entire Sub-Saharan Africa (SSA) region (Afinotan 2022). In addition, air pollution and extreme heat in Nigeria frequently exceed WHO guidelines, disproportionately affecting children, women, older adults, and socially disadvantaged groups (Rim-Rukeh 2015). Much of this pollution stems from the widespread use of biomass fuels (wood, charcoal) in poorly ventilated kitchens for cooking (Adeloye et al. 2013). Household air pollution alone accounts for over half of childhood deaths from acute respiratory infections, while continuous exposure to indoor and outdoor pollutants has driven up asthma prevalence among children (WHO and UN FCCC 2015; Awofeso 2011).

Addressing the link between climate change and respiratory health in Nigeria would require in-depth research, weather monitoring, policy reforms and effective adaptation and mitigation strategies. This is particularly true in vulnerable communities, such as the low-income urban areas of fast-growing cities like Lagos, Kano, Abuja, Ibadan, Port Harcourt, Warri, and Enugu, which are disproportionately affected by climate-related health hazards (Adelekan and Fregene 2015; Oni et al. 2021). Despite some national efforts, including the establishment of the Climate Change Act (CCA), National Council on Climate Change (NCCC), National Adaptation Strategy and Plan of Action on Climate Change and the Nigerian Climate and Health Observatory (Onyeneke et al. 2020), gaps in implementation have been reported (Afinotan 2022; FME Nigeria 2021). These gaps notably include insufficient funding, limited stakeholder engagement (particularly at the community level), inadequate enforcement mechanisms, and fragmented coordination among different governmental agencies (Asekun-Olarinmoye et al. 2014). Experts have opined that Nigeria would require improved community action and integrated climate change and public health policies to tackle climate risks (Asekun-Olarinmoye et al. 2014). This highlights the importance of our study. We aimed to review (i) the landscape of climate change research, policies, programs, and events at the national and subnational levels in Nigeria, and (ii) explore how they impact the population and/or respiratory health to guide appropriate responses in the country.

## 2 Methods

### 2.1 Research question

(i) What are the existing research, policies, programs and events on climate change in Nigeria?
(ii) What are the reported impacts on respiratory and/or population health in Nigeria?

### 2.2 Guideline

At the initial phase, our study was guided by the Cochrane Rapid Reviews Methods Group guidelines, for which we developed a protocol (unpublished and unregistered) (Garritty et al. 2021; Haby et al. 2016). Following further searches and reviews, we then employed a scoping review approach using the Preferred Reporting Items for Systematic reviews and Meta-Analyses extension for Scoping Reviews Extension for Scoping Reviews (PRISMAScR) checklist to capture more studies and reports (Tricco et al. 2018) (Table S1).

### 2.3 Information sources and search strategy

Searches were conducted on January 10, 2024, with search dates set from 01 January 2000 to 09 January 2024. The databases searched included PubMed, African Journals Online (AJoL), and Google Scholar. We also searched official government websites, data repositories of the United Nations and the World Bank, media reports and newspaper articles for relevant information.

Across all searches, we utilised broad search terms such as “climate change,” “policies,” and “respiratory health,”. Specific terms were employed on scientific databases. The details on PubMed are provided in Table 1. Returned articles and documents from the databases were exported to the EndNote 21 version (Clarivate: Philadelphia, PA) for screening.

## 3 Selection criteria

Articles were screened by title, abstract (when provided) and full text. We included studies for data extraction based on the following criteria:

(1) Type of studies or documents.
  (i) original community-based studies,
  (ii) government reports, policies, proclamations.
  (iii) submissions from relevant ministries. media reports, and newspaper articles.
(2) Study focus.
  (i) studies or documents must provide information related to the impacts of climate change on respiratory and/or.
  (ii) information on the impacts of climate change on population/public health in Nigeria.
(3) Case definitions of keywords– climate change, respiratory health and population health.
  (i) climate change: studies that explore temperature changes, extreme weather events, air quality, or greenhouse gas emissions as indicators of climate change.
  (ii) respiratory health: studies examining respiratory conditions like asthma, chronic obstructive pulmonary disease (COPD), lung cancer, or respiratory infections affected by climate, weather and environmental factors.
  (iii) population health: studies on the broader health impacts on communities or populations, including morbidity, mortality, and overall well-being in relation to climate conditions.

### 3.1 Data items and extraction

Two investigators (VOO and FNO) independently conducted screening and data extraction. Disagreements between reviewers were resolved by consulting a third reviewer (DA). A purpose-built extraction form was employed for data extraction and stored in Microsoft Excel file format. Key items extracted from studies included study characteristics, sector-level climate events, respiratory/population health impacts, and policies/programs reports and/or recommendations (Table S2).

### 3.2 Critical appraisal of individual studies

As we included reports and grey documents that were not necessarily research articles, we did not conduct any critical appraisal or quality assessment of the studies, documents or reports included in this review.

### 3.3 Data synthesis and analysis

We adopted a sector-level framework based on the World Health Organization (WHO) guidelines to synthesise data (Fekete et al. 2021; WHO 2023). The framework (Box S1) covered important sectors, including electricity generation, fossil fuel extraction, cleaner energy subsidies, manufacturing, construction, transportation, agriculture, land use and forestry, and waste management. First, we sorted extracted studies and documents by sector level and synthesised findings along the sectors. Second, we reviewed reported policies and programs relevant to climate change and respiratory and/or population health across studies and highlighted gaps and areas requiring further policy action. We also explored the inter-play between different sectors (e.g., the influence of electricity generation on agricultural practices). Finally, we examined the population’s adaptive strategies to mitigate impacts and the roles of key stakeholders in climate policy and action, with particular reference to respiratory and/or population health.

## 4 Findings

### 4.1 Search results

**Fig. 1:**
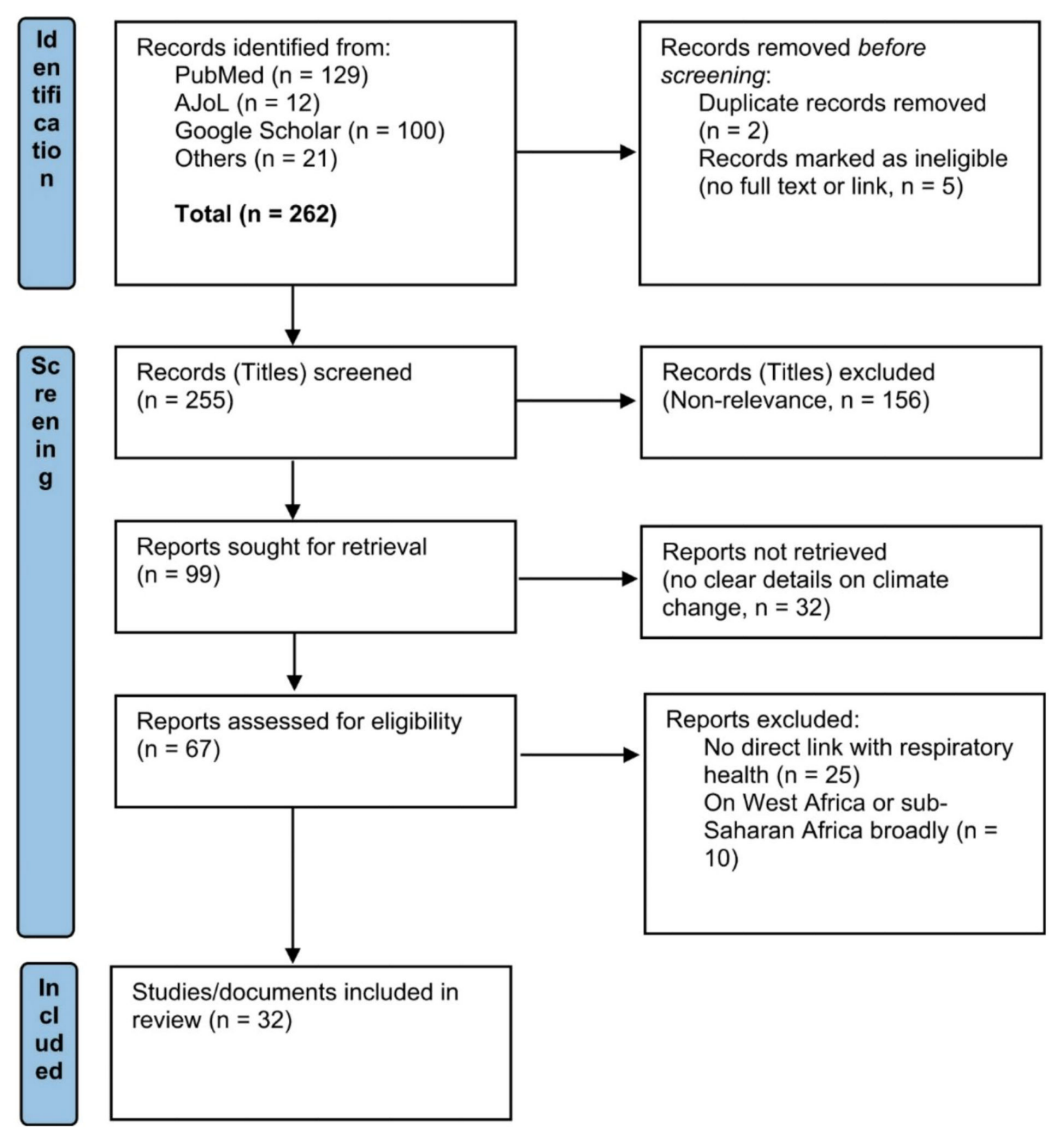
PRISMA flow chart of study selection process

Our searches yielded 262 studies and documents. These comprised 129 studies from PubMed, 12 from AJoL, the first 100 from Google Scholar (where saturation was reached), and an additional 21 documents from other sources, including grey literature, newspapers, reports, and official websites. Following the application of the selection criteria (i.e., relevance for climate change and respiratory health in Nigeria), a final list of 32 (studies and documents) that met the selection criteria were retained for extraction, review and synthesis (see Fig. 1 for details of study selection).

### 4.2 Study characteristics

Most of the studies/documents selected were nationwide and/or opinion-based reports, reviews or commentaries on climate change (20, 62.5%). Original population or community-based studies accounted for 21.9% (7 articles), and specific country sector analysis of climate change accounted for 12.5% (4 articles). Only one newspaper article was included (3.2%). Most of the articles reported findings from Lagos State and the Niger Delta region, in the southwest and south-south geopolitical zones of Nigeria, respectively.

### 4.3 Sector-specific synthesis and insights from individual studies

#### 4.3.1 Electricity generation and fossil fuel extraction

In 2020, greenhouse gases (GHG) in Nigeria totalled 126.9 million tonnes, with the energy sector accounting for the largest source of GHG emissions (60% of total emissions) (Ladan 2022). The detrimental effects of inadequate energy sector regulation and links between fossil fuel emissions and respiratory health issues were underscored across multiple studies (Table 2). For example, Afinotan (2022) reported high emissions from poorly regulated gas flaring activities in the Niger Delta region, while Akhionbare and Osuji (2013) linked these practices to increased respiratory diseases and social unrest in affected communities. Additional studies by Aye and Wingate (2019), Okon et al. (2021a), Olujobi (2020), and Urhie et al. (2020) echoed these findings, emphasising substantial health con, significant revenue losses, and biodiversity impacts. They noted the urgent need for policy reforms, while advocating for the diversion of flared gas to cooking and electricity. This will involve developing gas infrastructure and promoting a transition to cleaner energy sources to protect health and support economic growth without compromising environmental integrity.

#### 4.3.2 Subsidies for cleaner energy

The relationship between household energy sources and respiratory illnesses was reported by Atubi (2015), Awofeso (2011), and Fakinle et al. (2020). They particularly noted how indoor pollution from biomass and fossil fuel combustion significantly affects respiratory health, particularly among women and children. Obaseki et al. (2017) reported no significant effect on the prevalence of chronic airflow obstruction among respondents in their population-based study. Still, they noted that solid fuel for domestic cooking or heating was associated with a higher risk of respiratory symptoms, like cough, sneezing or phlegm. In addition, Raimi et al. (2021a). further illustrated the broad effects of climate change on household health, with the UN Nigeria country report specifically noting the indirect impact of heatwaves and carbon emissions on respiratory conditions (WHO and UN FCCC 2015).

Authors called for the promotion of renewable energy subsidies, like the domestic renewable heat incentives, to assist households to mitigate climate-related health risks (Table 3).

#### 4.3.3 Manufacturing, building, and transport

Studies by Atubi (2015), Croitoru et al. (2020) Fakinle et al. (2020), Mustapha et al. (2011) and Olalekan et al. (2018) explored the critical air quality challenges posed by emissions from transportation, industry, and construction. These sectors were identified as major contributors to the high levels of pollutants. These include particulate matter (PM) 2.5, PM10, carbon monoxide (CO), sulphur dioxide (SO2), nitrogen oxides (NOx), and volatile organic compounds (VOCs), which are closely associated with various respiratory conditions.(Atubi 2015; Croitoru et al. 2020; Fakinle et al. 2020; Mustapha et al. 2011; Olalekan et al. 2018) They underscored the importance of strengthening environmental regulations, particularly for vehicles and urban transportation, to improve urban air quality and protect public health (Table 4).

#### 4.3.4 Agriculture, land use/forestry, and waste management

Environmental degradation arising from agricultural practices, mineral exploitation, and waste management has both direct and indirect implications for respiratory health. In a newspaper review (Ozoh 2018), a large population of local residents were exposed to high levels of health-damaging pollutants and were at risk of adverse respiratory health outcomes due to months of continuous burning of Olusosun dump site in Lagos (Table 5). These are also in keeping with the findings of Adetona et al. (2020). Studies by Ajala (2018), Durodola (2019), Omotehinse and Ako (2019), Onah et al. (2016), and Pona et al. (2021) discussed the environmental and health consequences of agricultural, land use and forestry practices. For example, pollutants from plank and sawmills, as well as poultry and animal dung, significantly contribute to respiratory illnesses through emissions of particulate matter, VOCs, ammonia (NH_3_), hydrogen sulfide (H_2_S), Methane (CH_4_), carbon dioxide (CO_2_) and microbial contaminants. They noted fine wood dust and VOCs from sawmills cause airway inflammation and allergic reactions, exacerbating asthma and chronic respiratory issues, while NH_3_ and H_2_S from animal waste lead to mucosal irritation, lung function decline, and increased respiratory infections. They suggest a need for robust environmental laws, waste management, dust control systems, personal protective equipment and stricter regulations to reduce exposure.

#### 4.3.5 Climate change events and health impacts

Adeboyejo and Adewoyin (2017), Ajibade et al. (2013), Aliyu and Bota (2018) Echendu (2020), and Ilevbare (2019) explored the direct respiratory health impacts of climate change events. They specifically reported on increased respiratory diseases among the elderly due to fluctuating weather conditions and the exacerbation of respiratory symptoms from urban pollution. Authors call for adaptive healthcare strategies, inclusive public health policies, and improved environmental and urban management to mitigate the health impacts of climate change (Table 6).

#### 4.3.6 Policy and program analysis

Ladan (2022) reported that Nigeria’s 2021 CCA is the first stand-alone comprehensive climate change legislation in West Africa, which demonstrates Nigeria’s readiness to approach climate action and advocacy. The CCA provides a framework for mainstreaming climate actions in line with national development priorities and a net-zero target for 2050–2070 (Olujobi 2024). Prior, the National Environmental Standards and Regulations Enforcement Agency (NESREA) Act was established in 2007 to protect the environment through the enforcement of environmental laws, guidelines, and regulations in Nigeria, including implementing international agreements, protocols, and treaties on the environment that Nigeria has signed (Ladan 2022; FGN 2007). In the 2024 29th UN Climate Change Conference (COP 29) in Baku, Azerbaijan, President Bola Tinubu noted the approval of the Climate Accountability and Transparency Portal (CATP), alongside additional measures aimed at enhancing efficiency and accountability (State House Nigeria 2024). Furthermore, he authorised a comprehensive restructuring of the NCCC and the Intergovernmental Committee on the National Carbon Market Activation Plan (NCMAP) (State House Nigeria 2024). Across many studies (Aye and Wingate 2019; Anaebo and Ekhator 2015; Olujobi 2020; Olashore 2019; Babatunde 2010), authors noted there are policies and government proclamations aimed at mitigating the impact of climate change, but their relevance to respiratory or population health is not clearly documented (Table 7). Ladan (2022) reported there are several necessary measures needed to operationalise the CCA, including those related to addressing the health impacts of climate change, while the NESREA has been reported to be lacking in one of its main objectives to coordinate with stakeholders on environmental enforcement. It also remains unclear how this act addresses the oil and gas sector, particularly the fight against continued gas flaring and environmental degradation (Ladan 2022; FGN 2007).

Meanwhile, efforts to mitigate the environmental impact of oil extraction, particularly gas flaring in the Niger Delta, have largely been unsuccessful. For example, Aye and Wingate (2019) highlights persistent non-compliance with anti-flaring legislation, pointing to the ineffectiveness of policies such as The Associated Gas (Reinjection) Act (AGRA) of 1997 and The Flare Gas (Prevention of Waste and Pollution) Regulations. The continuous disregard for such regulations has led to continued pollution and health issues. Babatunde (2010) provides a broader perspective on government programs, critiquing their limited success due to a focus on income over human-centric development. Despite the CCA, the absence of a clearly defined implementation framework to address climate change is a critical gap identified (Olashore 2019; Olujobi 2024). This is also applicable to existing national programmes like the National Adaptation Strategy and Plan of Action on Climate Change and the Nigerian Climate and Health Observatory.(Onyeneke et al. 2020) This gap has allowed the impacts of climate change to go unmitigated. Olujobi (2020) presents Nigeria as a paradoxical figure: a top oil producer that simultaneously suffers from the ill effects of unregulated gas flaring. They underscore the pressing need for the Nigerian government to improve the domestic gas network, promote the use of liquefied petroleum gas (LPG), and enforce anti-flaring laws more rigorously to curb pollution and its health impacts.

### 5 Discussion

### 5.1 Summary of findings

This review is the first to comprehensively examine research, policies, and programs around climate change and respiratory health in Nigeria. Respiratory pollutants in the country—such as particulate matter (PM2.5, PM10), gaseous emissions (CO, SO_2_, NOx), agricultural by-products (NH_3_, H_2_S), greenhouse gases (CH_4_, CO_2_), and microbial contaminants—are linked to respiratory symptoms and allergies, inflammation and infections, and chronic conditions like wheezing, asthma, and COPD. These pollutants primarily stem from (i) gas flaring, predominant in the Niger Delta and South-South regions, (ii) high vehicle emissions in urban cities like Lagos and Kano, (iii) indiscriminate burning of municipal wastes, and (vi) widespread reliance on biomass for household cooking and heating.

Although the Climate Change Act (CCA) of 2021 marks a significant step as Nigeria’s first comprehensive climate legislation, it lacks clear directives on addressing respiratory or population health impacts, and the framework for its implementation remains underdeveloped. Additional legislations, such as the 1997 AGRA, 2007 NESREA, and the Flare Gas Regulations, also reveal critical implementation gaps, including limited enforcement, inadequate monitoring, and lack of community-level engagement. Our review highlights the need to enhance the enforcement of environmental laws, boost investments in renewable energy, and adopt comprehensive public health measures to mitigate these impacts.

### 5.2 Findings in the context of the wider literature

Our findings on climate change and respiratory health impacts underscores the role of climate change in exacerbating respiratory diseases (Adeloye et al. 2022; Song et al. 2022). Zhang et al. (2018) found a significant association between long-term PM2.5 exposure and elevated COPD mortality. This pattern is likely to extend to Nigeria, where air quality is deteriorating due to rapid industrialisation, urban expansion, and relaxed environmental regulations (Adekunle et al. 2018; Akpodiogaga-a and Odjugo 2010). Air pollutant levels in major urban areas such as Lagos and the Niger Delta exceed Federal Environmental Protection Agency (FEPA) standards, posing substantial health risks to the local population (Adeboyejo and Adewoyin 2017; Ajibade et al. 2013; FME Nigeria 2021). Climate-induced flooding, which is common in Lagos, poses further risks to respiratory health for vulnerable populations, particularly women, children, and the elderly (Ajibade et al. 2013; Echendu 2020). Flooding events lead to stress, increase indoor air pollution, promote mould growth, and raise exposure to respiratory pathogens, worsening conditions like asthma, respiratory infections, and chronic respiratory diseases. Additionally, women face disruptions in livelihoods and limited access to healthcare, intensifying their health risks. At the same time, children and the elderly are especially susceptible due to their higher vulnerability and limited mobility in emergencies (Ajibade et al. 2013; Echendu 2020). Despite infrastructure efforts by the Lagos State Government to protect neighbourhoods, economically disadvantaged and flood-prone communities are often left to develop their personal coping mechanisms (Oni et al. 2021; Pona et al. 2021).

Rising mean annual temperatures across Nigeria is another climate-related factor influencing respiratory health (Eludoyin et al. 2014; Michael and Dankyau 2022; Song et al. 2021). Higher temperatures, especially in urban cities such as Lagos, Ibadan, Kano, Abuja, and Port Harcourt, have been reported and contribute to increases in respiratory and cardiovascular conditions.(Adeboyejo and Adewoyin 2017) While some studies report conflicting data on the extent of temperature rise (Akpodiogaga-a and Odjugo 2010; Omoruyi and Kunle 2012), policy responses have been insufficient to address these health impacts effectively.

The management of Nigeria’s gas-flaring practices highlights a fragmented and inefficient regulatory framework that lacks cohesion and enforcement (Olujobi 2020). Interviews from Abuja indicate a gap in climate change awareness and policy at subnational levels, supporting findings from the Niger Delta, where gas flaring continues to degrade environmental and health conditions (Kaur and Pandey 2021; Muindi et al. 2023; Olashore 2019). Additionally, reliance on biomass for household energy exacerbates household air pollution and respiratory issues, particularly among women and children in rural areas and suburban slums (Adeloye et al. 2022; Ale et al. 2022). This dependence on biomass remains a serious health concern in African contexts, reinforcing the need for cleaner energy solutions.

Our review also highlights the impact of traffic pollution on respiratory health. Congested roads and ageing vehicles contribute to high PM2.5 levels, worsened by the use of imported fuels with high sulfur content (Atubi 2015; Mustapha et al. 2011). While the Intended National Determined Contribution (INDC) Policy proposes limits on older car imports, enforcement remains challenging due to disorganised port systems in Nigeria (FGN 2021). Lagos is exploring vehicle scrapping initiatives and expanding its transportation projects to reduce emissions (Atubi 2015; Croitoru et al. 2020). With many studies linking traffic pollution to respiratory symptoms, particularly in children, comprehensive policy measures are essential to mitigate these health impacts (McDuffie et al. 2021; Perez et al. 2013).

As noted, agricultural practices, land misuse, and biodiversity loss also significantly impact respiratory health in Nigeria. Intensive farming methods, deforestation, and unsustainable land use have led to severe biodiversity loss, soil degradation, and increased particulate matter in the air, worsening respiratory conditions (Durodola 2019; Omotehinse and Ako 2019). Practices like slash-and-burn agriculture release particulates and carbon into the atmosphere, reducing air quality and increasing respiratory health risks (Eludoyin et al. 2014; WHO and UN FCCC 2015). For example, the persistent burning of agricultural waste and land-clearing in rural areas releases pollutants that affect nearby urban centers, compounding urban air quality issues (Durodola 2019; Omotehinse and Ako 2019).

Moreover, as urbanisation expands into previously undeveloped areas, it displaces natural habitats and reduces green spaces that are crucial for filtering air pollutants (Durodola 2019). This expansion, without adequate environmental planning, contributes to habitat destruction, which not only harms biodiversity but also reduces the natural regulation of air quality, exacerbating respiratory health risks for urban and rural populations alike (Omotehinse and Ako 2019). In Lagos, informal settlements often encroach on wetlands, which are natural buffers against floods and air pollutants. These encroachments disrupt ecosystems and lead to a higher incidence of flooding, mold, and indoor air pollution, directly impacting the respiratory health of residents (Ajibade et al. 2013; Echendu 2020).

We also reported poor waste management practices, particularly open burning of waste, as a major contributor to poor air quality and respiratory health problems in many settings. A notable example is the Olusosun dumpsite fire in Lagos, which burned for over two months, releasing toxins that severely affected the respiratory health of residents (Adetona et al. 2020; Ozoh 2018). The failure to manage waste sustainably exacerbates air pollution, particularly in densely populated areas where improper waste disposal and burning release significant levels of particulate matter and other harmful pollutants.

Nigeria’s climate policies reveal a lack of effective implementation and enforcement, which affects climate mitigation and adaptation (Afinotan 2022; Okoye et al. 2022; Onyeneke et al. 2020). While various administrations have acknowledged environmental issues like gas flaring, weak political resolve and inconsistent law enforcement undermine progress (Okoye et al. 2022; Olujobi 2020; Okon et al. 2021b). Although recent actions like the establishment of the CCA, NCCC and CATP signal potential improvement, Nigeria still appears to lack a dedicated framework for climate change response and action, which is critical for assessing the effectiveness of climate and health initiatives (Afinotan 2022; Ladan 2022).

### 5.3 Study limitations

First, we did not conduct any quality assessment or critical appraisal of the studies in this review, given the broad nature of sources and document types. Research studies are limited, particularly the paucity of data from long-term studies that directly link respiratory health outcomes with climate change and policies, which has important research and policy response implications. We also note that we could not conduct quantitative analysis or even provide simple statistical summaries given the challenges in data consistency, regional focus (most studies are from Lagos and Niger Delta), and varied methodologies. Additionally, our review does not take into account the broader socioeconomic influences on policy effectiveness, such as demographic factors and local economic conditions. We also note that studies on the health impacts of exposure to heat and extreme thermal conditions are very limited in Nigeria, as most studies retained focussed primarily on air pollution. Despite these limitations, our study is the first detailed research paper on climate change and respiratory health in Nigeria, and indeed in sub-Saharan Africa, with several research, policy and practice implications in the region. It also highlights the critical social and health dimensions of environmental issues– a crucial consideration for policymakers, advocates and climate activists in the country.

### 5.4 Gaps identified and future directions

A critical gap in Nigeria’s current approach to climate and respiratory health lies in weak policy implementation. Existing policies often lack enforceable guidelines, particularly regarding air quality regulations, emissions controls, and land-use planning, which fall short in addressing the health impacts of pollution on the population. Limited coordination among regulatory bodies and insufficient funding further hinder the effective enforcement of these regulations. Additionally, gaps in long-term air quality and climate data monitoring prevent informed policy adjustments, limiting proactive responses to worsening environmental conditions.

To address these gaps, Nigeria requires a ‘*multi-layered policy approach’* that incorporates comprehensive air quality monitoring, research on climate-respiratory health linkages, and tailored health interventions addressing both respiratory and broader population health. This approach involves integrating various governmental levels and sectors to create cohesive policies that address emissions reduction, urban planning, and healthcare access. Specific interventions could include:

- **Health interventions**: Programs targeting respiratory health directly, such as improved access to respiratory disease management services, vaccination programs, and expanded national immunization efforts to mitigate infection-related respiratory complications. Health interventions should also include heat adaptation strategies, with public health systems prepared to address heat-related illnesses such as heat stroke, dehydration, and exacerbation of chronic conditions.
- **Public awareness campaigns**: Educational programs to raise awareness about the health impacts of air pollution and climate change, empowering communities with knowledge on preventive actions and available health resources.
- **Household and industrial pollution mitigation**: Enforcing stricter regulations on industrial emissions and supporting clean household energy alternatives (such as cleaner cooking fuels or solar solutions) to reduce indoor pollution and improve household air quality.
- **Adaptive health strategies**: With rising temperatures and rainfall variability, government and health systems should adapt to address the increased incidence of heat-related and vector-borne illnesses through early warning systems and heat health action plans. In Nigeria, this can involve using low-cost communication channels, public hydration campaigns, community outreach for heat illness prevention, shaded public cooling spaces, healthcare provider training for heat-related conditions, guidelines for outdoor work breaks during peak heat, and volunteer check-ins for vulnerable individuals. Moreover, there is a notable gap in research on the health impacts of extreme temperatures and heat exposure in Nigeria. Future research should also prioritise this area, as well as explore the socio-economic disparities that influence vulnerability to climate impacts.

To strengthen the evidence base for policy decisions, the establishment of a national respiratory health registry could provide critical data on trends and inform targeted interventions. Cost-benefit analyses of air quality improvement measures and partnerships with international organizations to learn from best practices are also necessary. Furthermore, longitudinal studies on the impact of sustained air quality interventions will provide insights into the long-term health and economic benefits, guiding more effective and adaptive policies.

Developing a community-focused model for climate and health initiatives is essential in Nigeria’s urbanising landscape. Rapidly growing cities like Lagos, Kano, Abuja and Ibadan would benefit from a dedicated air quality monitoring network that informs responsive policymaking and resource allocation. In urban planning, adopting *low-emission transport policies* to encourage the use of electric vehicles, promote non-motorized options (e.g., walking, cycling), and invest in alternative mass transport (rail and waterways) could reduce congestion and vehicular emissions, fostering a healthier urban environment.

Transitioning to clean energy sources is fundamental. Nigeria faces significant energy challenges, leading to dependence on diesel and fuel generators that contribute heavily to pollution. Developing cost-effective, sustainable pathways for clean energy adoption, supported by government incentives, will be crucial to mitigating air pollution and supporting the population’s respiratory health.

Legal amendments are also necessary, particularly making environmental violations enforceable under the Constitution and revising NESREA Act to encompass the oil and gas sector, thereby strengthening the fight against gas flaring and environmental degradation. Reducing and eventually halting gas flaring is a priority, with an emphasis on converting this natural gas into a cleaner energy source. This aligns with the need for a transition to domestic uses of cooking and industrial gases, including a shift towards centralised electricity that is both affordable and sustainable. Importing diesel engines that meet high emission reduction standards should become the norm. Governmental inaction and economic pressures further complicate Nigeria’s response to climate change. Instances of land misuse and biodiversity loss due to unchecked land acquisitions by foreign entities demonstrate the need for more accountable and ecologically responsible governance. Media suppression concerning government handling of climate issues and a reactionary stance towards natural disasters reflect inadequate strategic planning. Lastly, addressing the operational challenges within the Niger Delta Development Commission (NDDC) is crucial. By streamlining its responsibilities and minimising political interference, the NDDC can be transformed into a more effective agency that ensures responsible use of its resources for climate and related health risks in this region (Table S3).

## 6 Conclusions

This study addressed three key research gaps by mapping existing climate change research, policies, programs, and events in Nigeria (objective 1); examining how well these initiatives mitigate respiratory and population health impacts (objective 2); and identifying critical implementation shortfalls—such as weak enforcement and insufficient funding—that threaten public well-being to guide appropriate responses. Our findings underscore the urgent need for a more robust climate framework in Nigeria, including stronger policy enforcement, public education on health protection, and sustainable development approaches that integrate local cultural practices. Achieving these will require stricter environmental regulations, greater investment in clean energy, and concerted community-driven initiatives. We hope that this review can serve as an important resource for policymakers, researchers, and advocates in Nigeria and across Africa, guiding efforts to protect respiratory health and strengthen overall population well-being in an evolving climate crisis.

## Supplementary Material

The online version contains supplementary material available at https://doi.org/10.1007/s10584-025-03880-0.

Supplementary Information

## Figures and Tables

**Table 1:** Search terms

#	Searches	Hits
24	20 AND 21 AND 22 AND 23	129
23	15 OR 16 OR 17 OR 18 OR 19	10,821,682
22	9 OR 10 OR 11 OR 12 OR 13 OR 14	3,005,475
21	1 OR 2 OR 3 OR 4 OR 5 OR 6 OR 7 OR 8	268,246
20	“nigeria“[MeSH Terms] OR “nigeria“[All Fields] OR “nigeria s“[All Fields]	76,104
19	“policy“[MeSH Terms] OR “policy“[All Fields] OR “policies“[All Fields]	728,866
18	“activity“[All Fields]) OR “activity“[All Fields]	6,351,883
17	“program“[All Fields] OR OR “programme“[All Fields] OR “programmes“[All Fields] OR “programs“[All Fields]	1,952,291
16	“event“[All Fields] OR “event s“[All Fields] OR “events“[All Fields]	1,128,061
15	“legislation and jurisprudence“[MeSH Sub-heading] OR (“legislation“[All Fields] AND “jurisprudence“[All Fields]) OR “regulation“[All Fields]	3,397,618
14	“population health“[MeSH Terms]	1,016,591
13	breathing problems	13,321
12	“signs and symptoms, respiratory“[MeSH Terms] OR (“signs“[All Fields] OR “respiratory symptoms“[All Fields]	264,383
11	“respiratory tract diseases“[MeSH Terms] OR “respiratory disease“[All Fields] OR “respiration disorders“[MeSH Terms] OR (“respiration“[All Fields] AND “disorders“[All Fields]) OR “respiration disorders“[All Fields]	1,895,272
10	breathing disorders	98,730
9	respiratory health	227,477
8	“oil“[All Fields] AND (“exploration“[All Fields])	10,255
7	(“gas“[All Fields] AND (“flared“[All Fields] OR “flaring“[All Fields])	206
6	greenhouse gas	225
5	“floodings“[All Fields] OR “floods“[MeSH Terms] OR “floods“[All Fields]	27,800
4	(“extreme“[All Fields] AND (“rainfall“[All Fields] OR “rainfalls“[All Fields])	2,008
3	(“adverse“[All Fields] AND (“temperature“[MeSH Terms] OR “temperature“[All Fields]	45,106
2	“air pollution“[MeSH Terms] OR “air pollution“[All Fields]	103,270
1	“climate change“[MeSH Terms] OR “climate change“[All Fields]	92,056

Note: This search was conducted on PubMed, and was adapted for other searches

**Table 2:** Findings on electricity generation and fossil fuel extraction

Author, Year	Study Type, Location, Year	Main Findings	Impact on Respiratory Health	Policies/Programs Recommendations
Afinotan 2022	Report, National, 2022	Inadequate regulation of gas flaring contributes to climate change and GHG emissions; high emissions from electricity generation are noted.	Increased air pollution leads to respiratory issues.	Calls for clarification and strengthening of emission reduction initiatives.
Akhionbare and Osuji 2013	Population-based study, Imo state, 2013	Environmental and socio-cultural challenges due to fossil fuel extraction in Oguta.	Direct link to increased respiratory diseases.	Enforcement of fossil fuel extraction regulations; empowerment of local communities for sustainable development per Agenda 21.
Aye and Wingate 2019	Adaptive CC analysis, Niger Delta region, 2019	Over 60% of natural gas flared in Nigeria, causing significant revenue losses and environmental harm.	Health issues and reduced plant biodiversity from gas flaring.	Repurposing flared gas for cooking or electricity generation.
Okon et al. 2021a	Report/Commentary, National, 2021	Nigeria profits from oil and gas despite environmental pollution; reducing gas flaring can enhance sustainability.	Potential impact on respiratory health from dry gas production.	Development of infrastructure for converting natural gas to usable energy; elimination of gas flaring.
Olujobi 2020	Gas Flaring Framework Analysis, National, 2020	High rates of gas flaring due to weak enforcement of laws, lead to significant greenhouse gas (GHG) emissions and natural resource waste.	Major source of air pollution affecting climate and health.	Promotion of domestic gas usage and optimization of gas for electricity; enhancement of gas network; encouragement of liquefied petroleum gas (LPG) usage; gas pricing reform.
Urhie et al. 2020	Report, National, 2020	Electricity supply meets less than 20% of demand; reliance on generators contributes to GDP but increases air pollution.	Positive correlation between GDP growth and air pollution affecting health performance.	Reduction of carbon emissions; exploration of alternative energy sources.
Ladan 2022	Report/Commentary, National, 2022	2021 Climate Change Act: The first stand-alone comprehensive climate change legislation in West Africa, with potential to become a strategic tool for climate change action and advocacy in Nigeria. National Environmental Standards and Regulations Enforcement Agency (NESREA) Act: Established in 2007 to protect the environment, through the enforcement environmental laws, guidelines, and regulations in Nigeria	Greenhouse gases in Nigeria totalled 126.9 million tonnes, with the energy sector accounting for the largest source of GHG emissions (60% of total emissions). Author noted there are several necessary measures needed to operationalise the Act, including those related to health impacts of climate change	The Act provides framework for main-streaming climate actions in line with national development priorities and a net-zero target for 2050-2070. They are also in line with the 2021 Paris Agreement.

**Table 3:** Findings on subsidies for cleaner energy

Author, Year	Study Type, Location, Year	Main Findings	Impact on Respiratory Health	Policies/Programs Recommendations
Atubi 2015	Report/Review, National, 2015	Significant contribution of biomass burning and fossil fuel combustion to airborne pollutants.	Indoor air pollution linked to respiratory symptoms and reduced lung function, especially in women.	Advocacy for government subsidies for renewable energy; decarbonization through alternative fuels.
Awofeso 2011	Report/Review, National, 2011	Prevalence of asthma linked to petrol/diesel generator use in homes.	Rise in asthma prevalence from 2003 to 2006.	Strategies for affordable, sustainable electricity: low-emission diesel engines, liquefied gas replacement, and investment in renewables.
Fakinle et al. 2020	Report/Review, Lagos state, 2020	Increase in air pollutant emissions from combustion processes in Lagos.	Biomass burning indoors is linked to respiratory symptoms and reduced lung function in women.	Urgent reduction of pollutants from combustion processes.
Raimi et al. 2021	Review of environmental health impacts, National, 2021	Noticeable climate change effects in Nigeria; predictions of more frequent and severe heatwaves.	Heat-related illnesses such as heat exhaustion and stroke, pose risks to vulnerable communities.	Government to improve awareness of energy-related environmental degradation; build health workforce capacity; and create climate protection partnerships.
WHO and UN FCCC 2015	Climate Change Health Impacts Analysis, National, 2015	Agriculture and ‘other’ sectors were the largest contributors to carbon emissions in 2000; decline in air quality benefits by 2013.	Implied impact on respiratory health due to poor air quality.	Promotion of renewable energy usage by health sector; evaluation of health co-benefits from climate mitigation policies.
Obaseki et al. 2017	Population-based cross-sectional study, Ile-Ife, Osun State, 2017	Increased risk of respiratory symptoms and lower mental quality of life among respondents who use solid fuel for household cooking or heating.	Although no significant effect in the prevalence of chronic airflow obstruction was reported, using solid fuel for domestic cooking or heating was associated with a higher risk of cough or phlegm.	None.

**Table 4:** Findings on manufacturing, building, and transport

Author, Year	Study Type and Location, Year	Main Findings	Impact on Respiratory health	Policies/Programs Recommendations
Atubi 2015	Report/Review, National, 2015	Urban transportation policies in Nigeria tend to favour short-term development over long-term sustainability and eco-friendliness. However, in Minna, carbon monoxide (CO) emissions from traffic were measured at 5000 ppm, which is below the WHO limit of 20,000 ppm, attributed to lower traffic volumes and limited industrial activities.	Nigeria’s practice of importing used vehicles contributes to the worsening of respiratory health problems as high income countries address transport-related environmental issues.	The study recommends that Nigeria should enhance environmental regulations for urban transportation, aiming for a balance between current needs and future sustainability.
Croitoru et al. 2020	Report, Lagos state, 2020	Lagos suffers from high levels of air pollution due to road transport, industry, and power generation, exacerbated by vehicle density, outdated emissions technology, and high sulfur fuel use. Ambient PM_2.5_ exposure contributes to 11,200 premature deaths annually in Lagos, which is the highest number in West Africa. PM2.5-related deaths in Nigeria are at 23.8 per 100,000 people, surpassing West Africa’s average of 18.4 per 100,000.	Exposure to PM_2.5_ leads to significant health issues including respiratory and heart diseases, chronic bronchitis, and childhood respiratory infections.	Policy interventions are needed to encourage alternative transportation such as walking and cycling, the purchase of low-emission vehicles, the use of lower sulfur fuels, investment in solar power, and tree planting to mitigate emissions.
Fakinle et al. 2020	Report/Review, Lagos state, 2020	The Lagos Metropolitan Area Transport Authority (LAMATA) reported in 2002 that vehicular activities contribute to approximately 43% of the deteriorating air quality in Lagos.	Vehicle emissions are linked to respiratory health risks, with high levels of PM10, CO, SO2, NOx, and VOCs associated with asthma, respiratory infections, and chronic lung diseases.	The review calls for the projection of emission estimates for the next decade and advocates for vehicle efficiency programs.
Mustapha et al. 2011	Population-based cross-Sectional Survey, Niger Delta region, 2011	The study found a modest association between ambient air pollution from traffic and respiratory symptoms in schoolchildren, noting the effects of outdoor and indoor air pollution on children aged 7–14 years in low socioeconomic status areas of the Niger Delta. Traffic, distance from roads, and daily exposure to pollutants were considered.	Traffic pollution was modestly associated with respiratory symptoms in schoolchildren. High percentages of children reported pollution at school or home, with significant traffic disturbance and overcrowding.	/
Olalekan et al. 2018	Population-based, Kwara state, 2018	The study highlighted the contribution of human activities like fossil fuel burning in vehicles and industrial processes to the generation of aerosols. The mean Air Quality Index (AQI) for volatile organic compounds (VOCs) in the study area was 240.8 ppm, with PM2.5 at 56.15 μg/m^3^, PM10 at 28.81 μg/m^3^, lower explosive level (LEL) at 107.23%, and formaldehyde at 2570.65 mg/m^3^. These levels, according to the Federal Ministry of Environment standards, pose health risks, particularly to sensitive groups.	The high levels of VOCs, LEL, and formaldehyde pose significant health risks, especially for sensitive groups such as children, women, the elderly, and those with respiratory conditions like asthma.	Drastic efforts are needed to reduce air pollution in Kwara state, with particular focus on sensitive groups.

**Table 5:** Findings on agriculture, land use/forestry, and waste management

Author, Year	Study Type & Location, Year	Main Findings	Impact on Respiratory Health	Policy/Program Recommendations
Ajala 2018	Report, National, 2018	Large land acquisitions for agriculture by foreign entities impacted local communities.	Potential indirect impact on respiratory health via environmental changes.	Ensure environmental laws involve public participation and equitable treatment in foreign land investments.
Durodola 2019	Review, National, 2019	Climate events like droughts and floods will increasingly affect agriculture and food security.	Indirect impact on respiratory health due to changes in environmental conditions.	Develop resilient agricultural practices and improve land management to adapt to climate change.
Omotehinse and Ako 2019	Review, Plateau & Enugu state, 2019	Poorly managed mineral exploitation led to significant environmental degradation.	Direct health risks from pollution and contamination, potentially affecting respiratory health.	Implement minimum environmental standards for mineral exploitation and enforce strict sanctions for non-compliance.
Onah et al. 2016	Review, National, 2016	Climate change adversely affects agriculture, leading to food shortages and social conflict.	Climate changes affect water supply and increase disease vulnerability, indirectly impacting respiratory health.	Promote conservative environmental values and practices to mitigate the effects of climate change.
Pona et al. 2021	Review, National, 2021	Poor waste management practices lead to flooding and disease spread.	Respiratory infections linked to environmental factors were the leading causes of death.	Intensify efforts against illegal mining and implement robust waste management legislation.
Ozoh 2018; Adetona et al. 2020	News-paper/ Population-based cross-sectional study, 2018, Olusosun, Lagos state	From March 14, 2018, the Olusosun dump site burned for over 2 months with significant health impacts on the immediate community.	A large population of Lagosians were exposed to high levels of health-damaging pollutants and were at risk of adverse respiratory health outcomes.	Lagos State government declared the Olusosun dump site closed. Active, strategic and well-designed steps to address the adverse health effects on at-risk persons were advocated for.

**Table 6:** Findings on climate change events and health impacts

Author, Year	Study Type and Location, Year	Main Findings	Impact on Respiratory Health	Policies/Programs Recommendations
Adeboyejo and Adewoyin 2017	Review, Lagos state, 2017	Climate-related diseases among the aged were partially attributed (11.5%) to fluctuations in rainfall and temperature. High blood pressure was the most prevalent, followed by febrile illnesses like malaria and typhoid.	Respiratory diseases constituted 17% of the climate-related cases reviewed.	The study suggests that climate variations are not solely responsible for disease prevalence; social and economic factors also play significant roles. Recommendations include addressing broader social determinants of health beyond just climate change.
Ajibade et al. 2013	Population-based study, Lagos state, 2013	Gender alone does not determine vulnerability to climate events like flooding; low-income women, particularly in areas such as Badia, are severely impacted due to economic constraints and discriminatory policies.	Flooding had significant health impacts on women’s lives and livelihoods, including their respiratory health.	Policies should promote free access to maternal and childcare facilities and repeal discriminatory health policies to better support affected women.
Aliyu and Botai 2018	Population based, Mixed-Methods, Kaduna state, 2018	Levels of carbonmonoxide CO), sulphurdioxide (SO2), Particulate Matters PM_10_, and PM_2.5_ surpassed WHO/FEPA limits, ranking Zaria among the top five polluted cities globally for particulate matter.	Outdoor air pollution, especially PM _2.5_, had a substantial impact on respiratory health indicators like coughing and wheezing.	The findings call for Nigeria to develop public health strategies and interventions informed by evidence on the effects of air pollution.
Echendu 2020	Review, National, 2020	Conversion of agricultural lands to housing without proper planning increases flooding risks. Effective spatial planning and flood risk management strategies are essential.	The study does not directly link flooding to respiratory health but implies poor interagency coordination in mitigating climate impacts.	It is recommended that Nigeria adopt a more proactive approach by funding pre-flood mitigation and adaptation strategies, rather than just allocating funds post-floods.
Ilevbare 2019	Review, National, 2019	Vulnerable groups, including women, children, the elderly, and rural populations, are dis-proportionately affected by the health risks of climate change. This includes an array of illnesses such as cerebrospinal meningitis, and cardiovascular and respiratory illnesses.	Climate change increases the prevalence of allergic and cardio-respiratory diseases due to water and food shortages, heat stress, and secondary pollutants.	The government should enhance climate change awareness through training, workshops, and sensitisation programs, targeting vulnerable groups specifically.
Weli and Efe 2015	Review, Rivers state, 2015	A significant rise in temperature and rainfall in Port Harcourt has been recorded, correlating with an increase in malaria prevalence due to enhanced mosquito development and breeding sites. There are seasonal patterns in malaria cases corresponding to peak rainfall months.	The study indirectly suggests that high humidity and wet environments from increased rainfall could exacerbate respiratory conditions.	Recommendations include regular clearing of drains and environments, especially during peak rainfall months, distribution of mosquito nets, and health programs aimed at reducing mosquito prevalence.

**Table 7:** Findings on policies, programs and events

Author, Year	Study Type and Location, Year	Main Findings	Impact on Respiratory Health	Policy/Program Recommendations
Anaebo and Ekhator 2015	Environmental framework evaluation, National, 2015	The Nigerian government prioritizes economic gains from oil, often at the expense of the environment and public health. Nigeria ranks as the second-largest gas-flaring nation, contributing to the vulnerability of its citizens to respiratory issues.	High vulnerability to respiratory problems due to extensive gas flaring.	Adoption of legally enforceable environmental rights is recommended. Nigeria could emulate South Africa in integrating the right to a healthy environment into its constitution.
Aye and Wingate 2019	Analysis of Flare Gas Regulations, Niger Delta region, 2019	Attempts to mitigate gas flaring in the Niger Delta have failed due to persistent non-compliance by companies. Notable policies include The Associated Gas (Reinjection) Act (AGRA) of 1997 and The Flare Gas (Prevention of Waste and Pollution) Regulations, which have increased the penalties for gas flaring.	Prolonged gas flaring for over 30 years has led to severe air pollution, exacerbating respiratory health issues.	Urgent and comprehensive action is needed to address gas flaring in Nigeria. Enforcement of environmental preservation and economic development policies is crucial, especially in the Niger Delta’s oil producing communities.
Babatunde 2010	Population-based Mixed Method Study, Niger Delta region, 2010	Government anti-poverty programs in the Niger Delta have seen limited success, focusing on income-based rather than human-based development.	N/A	A recommendation to allocate 60% of the state’s 13% oil mineral derivation fund to OSOPADEC, as opposed to the current 40%, to improve human-based development outcomes.
Olashore 2019	National Gas Flaring Framework Analysis, 2019	There is an absence of clear, actionable national legislation on climate change in Nigeria. While several acts, such as the Energy Commission Act and the Associated Gas Re-injection Act, have been passed, their implementation is hindered by a lack of energy laws and reliance on fossil fuels. The NESREA Establishment Act has led to environmental regulations, but the agency faces challenges like limited autonomy.	Climate change has a gradual yet significant socioeconomic and health impact on the Nigerian population.	Effective climate change legislation in Nigeria requires more than just laws; it necessitates public information and environmental education to raise awareness and enact change.
Olujobi 2020	National Gas Flaring Framework Analysis, 2020	Nigeria’s weak enforcement of anti-gas flaring laws has led to it becoming a top producer of crude oil with high rates of gas flaring. In 2018, Nigeria ranked 7th globally for gas flaring. This has led to significant greenhouse gas emissions and the loss of potential natural resource revenue.	Gas flaring, accounting for about 12.50% of the world’s total in 2006, is a major source of air pollution, with significant health impacts.	The Federal Government of Nigeria (FGN) is encouraged to promote the domestic use of cooking and industrial gases, enhance the gas network to boost the domestic market and encourage the optimisation of liquefied petroleum gas (LPG) usage. Gas pricing reforms are needed to attract more investment.

## Data Availability

Not Applicable.
