# Supplementary material for: Exploring the impact of climate change on respiratory health in Nigeria: a scoping review of current research, government policies and programs
Source: Clim Change. Author manuscript; Available in PMC 2025 Feb 25. (PMC11832684; doi:10.1007/s10584-025-03880-0)
Supplement: Supplementary Information [file EMS203433-supplement-Supplementary_Information.docx]

## Supplementary Information

## Exploring the impact of climate change on respiratory health in Nigeria: a scoping review of current research, government policies and programs

**Faatihah Niyi-Odumosu^1^, Obianuju B. Ozoh^2^, Victor Oloruntoba Ope^3^, Boni M. Ale^4,5,6,7^, Olayemi Akinnola^8^, Alexander Iseolorunkanmi^9^, Davies Adeloye^10*^ on behalf of the C^2^REST Nigeria Study**

1. School of Applied Sciences, University of the West of England, Bristol, UK
2. Department of Medicine, Faculty of Clinical Sciences, College of Medicine, University of Lagos, Lagos, Nigeria
3. Department of Sociology, Covenant University, Ota, Nigeria
4. Cardiovascular Research Unit, University of Abuja and University of Abuja Teaching Hospital, Gwagwalada Abuja, Nigeria
5. Institute of Tropical and Infectious Diseases, University of Nairobi, Nairobi, Kenya
6. Holo Global Health Research Institute, Nairobi, Kenya
7. Health Data Acumen, Nairobi, Kenya
8. Department of Biological Sciences, Covenant University, Ota, Nigeria
9. Covenant University Medical Centre, Covenant University, Ota, Nigeria
10. School of Health & Life Sciences, Teesside University, Middlesbrough, UK

***Correspondence:**

Dr Davies Adeloye

School of Health & Life Sciences, Centuria Building

Teesside University, Middlesbrough, TS1 3BX, UK

[D.Adeloye@tees.ac.uk](mailto:D.Adeloye@tees.ac.uk)

**Funding**

This study was funded by the Medical Research Foundation, United Kingdom (Grant reference: MRF-RG-ICCH-2022-100054). The funder had no role in the conduct and writing of the study.

**Competing Interests**

The authors declare no conflicts of interest.

**Table S1. Preferred Reporting Items for Systematic reviews and Meta-Analyses extension for Scoping Reviews (PRISMA-ScR) Checklist**

| **SECTION** | **ITEM** | **PRISMA-ScR CHECKLIST ITEM** | **REPORTED ON PAGE #** |
| --- | --- | --- | --- |
| **TITLE** | | | |
| Title | 1 | Identify the report as a scoping review. | 1 |
| **ABSTRACT** | | | |
| Structured summary | 2 | Provide a structured summary that includes (as applicable): background, objectives, eligibility criteria, sources of evidence, charting methods, results, and conclusions that relate to the review questions and objectives. | 1 |
| **INTRODUCTION** | | | |
| Rationale | 3 | Describe the rationale for the review in the context of what is already known. Explain why the review questions/objectives lend themselves to a scoping review approach. | 2 |
| Objectives | 4 | Provide an explicit statement of the questions and objectives being addressed with reference to their key elements (e.g., population or participants, concepts, and context) or other relevant key elements used to conceptualize the review questions and/or objectives. | 2 |
| **METHODS** | | | |
| Protocol and registration | 5 | Indicate whether a review protocol exists; state if and where it can be accessed (e.g., a Web address); and if available, provide registration information, including the registration number. | 3 |
| Eligibility criteria | 6 | Specify characteristics of the sources of evidence used as eligibility criteria (e.g., years considered, language, and publication status), and provide a rationale. | 3 |
| Information sources* | 7 | Describe all information sources in the search (e.g., databases with dates of coverage and contact with authors to identify additional sources), as well as the date the most recent search was executed. | 3 |
| Search | 8 | Present the full electronic search strategy for at least 1 database, including any limits used, such that it could be repeated. | 3, Table 1 |
| Selection of sources of evidence† | 9 | State the process for selecting sources of evidence (i.e., screening and eligibility) included in the scoping review. | 3 |
| Data charting process‡ | 10 | Describe the methods of charting data from the included sources of evidence (e.g., calibrated forms or forms that have been tested by the team before their use, and whether data charting was done independently or in duplicate) and any processes for obtaining and confirming data from investigators. | 3 |
| Data items | 11 | List and define all variables for which data were sought and any assumptions and simplifications made. | 3 |
| Critical appraisal of individual sources of evidence§ | 12 | If done, provide a rationale for conducting a critical appraisal of included sources of evidence; describe the methods used and how this information was used in any data synthesis (if appropriate). | 3 |
| Synthesis of results | 13 | Describe the methods of handling and summarizing the data that were charted. | 3-4 |
| **RESULTS** | | | |
| Selection of sources of evidence | 14 | Give numbers of sources of evidence screened, assessed for eligibility, and included in the review, with reasons for exclusions at each stage, ideally using a flow diagram. | 4, Figure 1 |
| Characteristics of sources of evidence | 15 | For each source of evidence, present characteristics for which data were charted and provide the citations. | 4 |
| Critical appraisal within sources of evidence | 16 | If done, present data on critical appraisal of included sources of evidence (see item 12). | 4 |
| Results of individual sources of evidence | 17 | For each included source of evidence, present the relevant data that were charted that relate to the review questions and objectives. | 5 |
| Synthesis of results | 18 | Summarize and/or present the charting results as they relate to the review questions and objectives. | 5-6 |
| **DISCUSSION** | | | |
| Summary of evidence | 19 | Summarize the main results (including an overview of concepts, themes, and types of evidence available), link to the review questions and objectives, and consider the relevance to key groups. | 6 |
| Limitations | 20 | Discuss the limitations of the scoping review process. | 7 |
| Conclusions | 21 | Provide a general interpretation of the results with respect to the review questions and objectives, as well as potential implications and/or next steps. | 7-10 |
| **FUNDING** | | | |
| Funding | 22 | Describe sources of funding for the included sources of evidence, as well as sources of funding for the scoping review. Describe the role of the funders of the scoping review. | 1, 10 |

JBI = Joanna Briggs Institute; PRISMA-ScR = Preferred Reporting Items for Systematic reviews and Meta-Analyses extension for Scoping Reviews.

* Where *sources of evidence* (see second footnote) are compiled from, such as bibliographic databases, social media platforms, and Web sites.

† A more inclusive/heterogeneous term used to account for the different types of evidence or data sources (e.g., quantitative and/or qualitative research, expert opinion, and policy documents) that may be eligible in a scoping review as opposed to only studies. This is not to be confused with *information sources* (see first footnote).

‡ The frameworks by Arksey and O’Malley (6) and Levac and colleagues (7) and the JBI guidance (4, 5) refer to the process of data extraction in a scoping review as data charting*.*

§ The process of systematically examining research evidence to assess its validity, results, and relevance before using it to inform a decision. This term is used for items 12 and 19 instead of "risk of bias" (which is more applicable to systematic reviews of interventions) to include and acknowledge the various sources of evidence that may be used in a scoping review (e.g., quantitative and/or qualitative research, expert opinion, and policy document).

*From:* Tricco AC, Lillie E, Zarin W, O'Brien KK, Colquhoun H, Levac D, et al. PRISMA Extension for Scoping Reviews (PRISMAScR): Checklist and Explanation. Ann Intern Med. 2018;169:467–473. [doi: 10.7326/M18-0850](http://annals.org/aim/fullarticle/2700389/prisma-extension-scoping-reviews-prisma-scr-checklist-explanation).

**Table S2. Data Extraction Template**


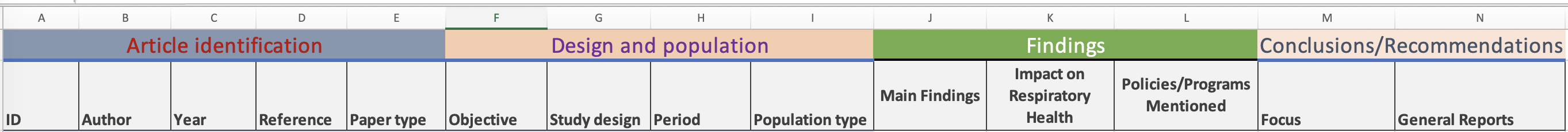


**Table S3. Sector-based recommendations for policy consideration**

| Sector | Key Recommendations | Rationale | Expected Outcomes |
| --- | --- | --- | --- |
| Electricity Generation and Fossil Fuel Extraction | Implement stricter emission controls and promote renewable energy sources | To reduce air pollution caused by emissions, which directly impacts respiratory health. | Improved air quality leading to decreased respiratory health issues. |
| Cleaner Energy Subsidies | Increase subsidies and incentives for renewable energy and cleaner technologies. | Encouraging the transition to cleaner energy sources can significantly reduce air pollution. | Reduced reliance on fossil fuels, leading to better air quality and health outcomes. |
| Manufacturing, Building, and Transport | Strengthen regulations on industrial emissions and promote sustainable urban planning. | High pollutant levels in these sectors contribute to poor air quality and respiratory problems. | Enhanced urban air quality and a healthier population, particularly in densely populated areas like Lagos, Abuja, and Kano. |
| Agriculture and Land Use | Promote sustainable agricultural practices and enforce deforestation laws. | Agricultural chemicals and deforestation affect air quality and climate patterns. | Preservation of natural resources, leading to improved air quality and reduced health risks. |
| Waste Management | Implement effective waste management systems, including recycling and proper waste disposal methods. | Poor waste management practices contribute to air pollution and respiratory health problems. | Better waste management leading to cleaner environments and reduced health risks. |
| Policy Implementation | Strengthen the enforcement and monitoring of existing environmental and health policies. | The effectiveness of policies is often hindered by weak implementation and lack of oversight. | Effective policy enforcement can lead to substantial improvements in public health. |
| Public Awareness and Education | Enhance public awareness campaigns on the health impacts of climate change. | Increased awareness can lead to community engagement and support for environmental initiatives. | A more informed public taking proactive steps to reduce personal and community health risks. |
| Research and Monitoring | Invest in ongoing research and monitoring of climate change's impact on health. | Continuous research is needed to understand and adapt to the evolving impacts of climate change. | Data-driven approaches to addressing and mitigating the health impacts of climate change. |

| **Box S1. Sector-Level Framework**  **Electricity Generation and Fossil Fuel Extraction**   - Policies and Programs: Identification of any initiatives or regulations targeting electricity generation and their implications for air quality and respiratory health.   **Subsidies for Cleaner Energy**   - Policies and Programs: Examination of policies regulating fossil fuel extraction and emission controls. - Initiatives: Overview of government subsidies for clean energy solutions.   **Manufacturing, Building, and Transport**   - Regulations: Assessment of environmental regulations in manufacturing, especially those related to emissions. - Building Codes: Exploration of green building codes and their impact on indoor air quality. - Policies: Review of transportation policies, including emission standards and promotion of public transport.   **Agriculture, Land Use/Forestry, and Waste Management**   - Agriculture: Analysis of agricultural practices adapted to climate change and their environmental impact. - Land Use/Forestry: Scrutiny of land use and forestry policies for climate adaptation and mitigation. - Waste Management: Assessment of waste management practices and policies.   Adapted from: (Fekete et al., 2021, Organization, 2023) |
| --- |

FEKETE, H., KURAMOCHI, T., ROELFSEMA, M., ELZEN, M. D., FORSELL, N., HöHNE, N., LUNA, L., HANS, F., STERL, S., OLIVIER, J., VAN SOEST, H., FRANK, S. & GUSTI, M. 2021. A review of successful climate change mitigation policies in major emitting economies and the potential of global replication. *Renewable and Sustainable Energy Reviews,* 137**,** 110602.

ORGANIZATION, W. H. 2023. Operational framework for building climate resilient and low carbon health systems. *Operational framework for building climate resilient and low carbon health systems.*
